# Influence of Periosteum on the Reproduction of Bone

**Published:** 1865-01

**Authors:** 


### INFLUENCE OF
### PERIOSTEUM ON THE REPRODUCTION OF BONE.

"The third meeting of the Medical Congress at Lyons was entirely devoted to the discussion of two surgical questions: 1. What surgical improvements have modern investigations on the osseous system effected? 2. On the value of the means which may advantageously be substituted for the knife with a view of preventing the accidents which result from wounds. The whole discussion on the first question was confined to the part which the periosteum plays in the regeneration of the osseous tissue. Here the two existing schools of surgery, the old and the modern, were in direct opposition: the former asserting the entire inactivity of the periosteum, and the utter inutility of preserving it in operations; the other attributing to that membrane the only active part in the regeneration of bones. M. Ollier, of Lyons, (who has acquired great reputation precisely on account of his numerous experiments on the periosteum, and the importance which he attaches to that membrane,) together with M. Verneuil, of Paris, warmly supported the latter opinion, while M. Desgranges and others attacked it.

"According to M. Ollier, the periosteum alone reproduces the bone; no other tissue can do the same. All bones may be reproduced, whatever their form. The condition of the general health of the patient is influential in the work of reproduction. This reproduction takes place in man as well as in the lower animals, clinical facts being here in accord with experimental facts: *hence* the necessity of sub-periosteal excisions—much easier and much more simple than the resection by the old system. In support of these assertions, M. Ollier presented to the Congress numerous anatomical specimens, the results of his different experiments. He showed, in cats, the radius and the peroneum reproduced in different parts where the periosteum had been preserved; whereas they were wanting where it had been taken away. In one specimen, which excited general admiration, the humerus of a dog was entirely reproduced with perfect regularity of form. M. Ollier exhibited short as well as long bones in which the osseous regeneration was visible. He insisted on the importance which should be attached to the influence of general hygienic conditions while considering the success or unsuccess following sub-periosteal operations.

"To these various arguments M. Desgranges answers: that experiments had been made by other *savants* which had proved unsuccessful; that it could not be supposed that a membrane wet with pus could produce the effects attributed to it; that if the periosteum can reproduce the osseous tissue in a rabbit such a fact has not as yet been clinically observed in man. And besides, why preserve the periosteum at all, when we are told that it can reproduce itself even when taken away? The periosteum, it had been said, tends naturally to attach itself to the bone; wherefors, then, the necessity that in amputations of limbs the extremity should be covered with the periosteum? If this were true, what a marvelous discovery it would be for surgery! There would be no inflammation; no suppuration of the bône. Unfortunately this was not the case. He had already tried it five times, and he had observed prolonged suppuration and greater difficulty to cure. The 'supporters of the periosteum' had extolled its utility in cases of comminutive fracture, but the Society of Surgery itself had lately discarded this notion. Besides, the term itself of 'sub-periosteal excision' was incorrect; for in Maisonneuve's cases and others, operations for simple necrosis bore that name. The true sub-periosteal excision would be rather the emptying of the bone proposed by M. Sédillot, of Strasburg. The true field for sub-periosteal excisions was to be found in osteoplastic operations, and hitherto they had proved unsuccessful. In conclusion, he saw no truth whatever in the new functions attributed to the periosteum.

"With these two discourses, which embody the best arguments on both sides, I leave the two schools, in presence. Let me not forget, however, to mention, as an important feature of the debate, the presentation made to the Congress by M. Aubert, a medical practitioner at Macon, of a young man, 17 years of age, on whom he had performed an eminently successful operation. This young man had been affected with a caries which ocupied the whole epiphysis of the left tibia, together with condensing osteitis occupying a little more than the lower third of the dyaphysis. M. Aubert made an incision in the skin; separated the periosteum, and that very easily, as it was inflamed and adhered but little to the bone; then, slipping a chain-saw beneath the periosteum, he took off all the diseased portion of the bone, and disarticulated the tibia at its lower extremity. Four years have elapsed since that operation, and the patient now possesses a tibia entirely regenerated, of the same form and volume as before. A very slight longitudinal depression scarcely indicates the part where the incision had been made. This newly-formed tibia ends below with an ankle perfectly formed; and the young man, whose health is vigorous, can walk fifteen miles a day, and dance during several hours, without any fatigue. This brilliant success, which speaks in favor of the sub-periosteal system, was saluted by the Congress with general applause."
—*Paris Correspondent of Lancet.*

